# Formation of Sodium Chloride on the Surface of Sulfate-Rich Gobi Desert Salt in Response to Water Adsorption

**DOI:** 10.1021/acsestair.4c00092

**Published:** 2024-10-17

**Authors:** Nicolas Fauré, Jie Chen, Luca Artiglia, Markus Ammann, Thorsten Bartels-Rausch, Zamin A. Kanji, Sen Wang, Jan B. C. Pettersson, Erik S. Thomson, Ivan Gladich, Xiangrui Kong

**Affiliations:** † Department of Chemistry and Molecular Biology, Atmospheric Science, University of Gothenburg, SE-41390 Gothenburg, Sweden; ‡ Department of Environmental Systems Science, ETH Zürich, Zürich, 8092, Switzerland; § Laboratory of Atmospheric Chemistry, Paul Scherrer Institute, CH-5232 Villigen PSI, Switzerland; ∥ Shaanxi Key Laboratory of Earth Surface System and Environmental Carrying Capacity, Northwest University, Xi’an 710127, China; ⊥ European Centre for Living Technology (ECLT), Dorsoduro, Calle Crosera, 30124 Venice, Italy; # Qatar Environment and Energy Research Institute, Hamad Bin Khalifa University, P.O. Box 31110, Doha, Qatar

**Keywords:** desert salt, water adsorption, ion migration, ambient pressure XPS, Gobi Desert, molecular dynamics, hygroscopicity

## Abstract

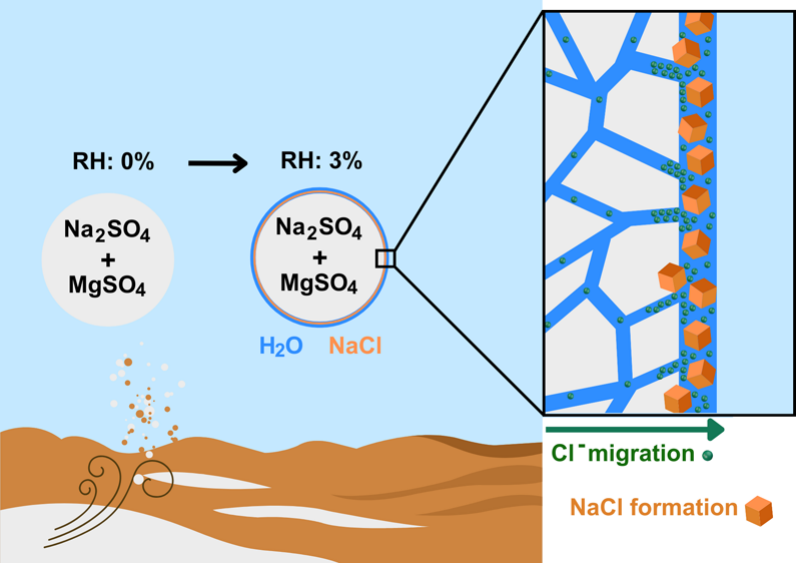

Dust storms in arid regions transport desert salts and dust, affecting geochemical processes, atmospheric chemistry, climate, and human health. This study examines how the gas–salt interface composition of desert salt changes with varying relative humidity (RH), using ambient pressure X-ray photoelectron spectroscopy (APXPS), near-edge X-ray absorption fine structure (NEXAFS) spectroscopy, and molecular dynamics (MD) simulations. Ion chromatography analysis of desert salt indicates it is predominantly composed of sulfate, sodium, and magnesium ions, with traces of calcium, chloride, nitrate, and potassium ions. APXPS and NEXAFS surface analyses show that, at 0% RH, the gas–salt interface primarily features Na_2_SO_4_, with smaller amounts of MgSO_4_ and a trace of NaCl on the top layers. As humidity increases, the composition at the gas–salt interface changes, notably with Mg^2+^ binding to SO_4_^2–^ ions and a dominant NaCl formation throughout the studied surface depth. This shift indicates a transition from a sulfate- to a chloride-rich surface as humidity increases, contradicting MD simulations that predicted that on salt crystals covered by a submonolayer of water with electrolytes, chloride ions migrate toward the liquid–solid interface. This discrepancy indicates that other factors, like enhanced ionic mobility at grain boundaries, might drive the accumulation of chloride ions at the gas interface. The study emphasizes the crucial role of adsorbed water in ion migration and surface composition transformation of desert salts, affecting geochemical processes in arid regions.

## Abstract 2

Water adsorption-driven transformation of Gobi Desert salts, leading to surface sodium chloride formation, with potential implications for atmospheric chemistry in dust storm-affected regions.

## 1 Introduction

Dust storms originated from arid areas such as the Gobi Desert, spanning northern China and southern Mongolia, whisk dust and salt particles from arid lakebeds, playas, and salt flats into the air.^1−3^ These mixtures of dust and salt particles can traverse extensive distances,^4,5^ and adversely impact air quality and potentially influence patterns of cloud formation^6−8^ and precipitation.^9−11^ Notably, during spring, the fierce dust storms of the Gobi Desert can markedly affect air quality and visibility across eastern Asia, including China,^12,13^ Korea,^13^ Japan,^13^ and even more remotely into North America^14^ and the Arctic.^15^

Naturally aerosolized salt particles are a common component in the atmosphere and significantly impact climate.^16^ They actively contribute to the formation of clouds due to their strong ability to absorb moisture. Salt particles or salt fractions in dust/salt mixtures can also alter the ability of aerosol particles to adsorb water vapor from surrounding environments, effectively serving as cloud condensation nuclei (CCN)^17^ and even ice nucleating particles (INP) in some conditions.^18^ Natural salts are often mixtures of different salt species and therefore have higher hygroscopicities than pure single component salts.^19^ The presence of soluble salt components can induce water uptake activation by fine dust aerosols that would not activate if completely insoluble in absence of salts.^10,23^

Beyond their role in hygroscopic processes, saline mineral particles catalyze a range of heterogeneous atmospheric chemical reactions.^20−24^ Such reactions are significant in regions like the North China Plain and the East Asian atmosphere, where saline mineral dust contributes to secondary aerosol formation, influencing haze events and air pollution dynamics.^21^ The reactivity of these particles with chemical compounds like nitrate and ammonia leads to the production of secondary compounds (e.g., NH_4_NO_3_), affecting atmospheric chemistry and human health.^20^ Laboratory studies have shown that these reactions can produce ClNO_2_ from playa dusts, suggesting the need to update models to include such sources for a more accurate representation of atmospheric chloride dynamics.^22^ Despite their importance, our understanding of the physicochemical properties of these mineral dust surfaces, especially in response to relative humidity (RH) changes, remains limited. This gap in knowledge is critical as over 40% of the world population resides in areas heavily affected by dust,^25^ emphasizing the need for a deeper understanding of the nature and behavior of these particles.

Focusing on the Gobi Desert, a significant source of East Asian dust storms, this study aims to refine our understanding of the surface properties on desert salt aerosol particles. Originating mainly from the saline lakes, salt flats, and playas around the desert,^5^ which face increasing threats due to climate change,^26^ these salt particles undergo wind lofting and transportation, contributing to the chemical and dynamic complexity of the desert’s atmospheric contribution. Previous research has analyzed the surface propensity of ionic species and the chemistry of pure^27−31^ and natural^32,33^ salts as water adsorbs, laying a foundational understanding of their behaviors and interactions. Building upon this groundwork, this study employs advanced surface-sensitive techniques to analyze the desert salt’s surface responses to RH changes. This investigation sheds light on the unique characteristics and environmental interactions of Gobi Desert salt aerosols. This provides valuable insights into the interaction between desert salts and their environment and improves our understanding of the formation and physicochemical properties of salt aerosol particles.

## 2 Material and Methods

In this study, we utilized two surface-sensitive methodologies, ambient pressure X-ray photoelectron spectroscopy (APXPS) and near-edge absorption fine structure (NEXAFS) spectroscopy, in conjunction with molecular dynamics (MD) simulations, to explore a natural salt sample collected from the Gobi Desert (42°17′50.6” N, 101°06′07.5” E). The experiments were carried out using only the soluble fraction of the Gobi Desert salt sample, and therefore contained no minerals. This approach prevents the formation of an inhomogeneous layer, which could introduce artifacts and compromise the accuracy of the measurements. Furthermore, X-ray photoelectron spectroscopy (XPS) is primarily sensitive to the topmost surface layer, making the soluble salt fraction most relevant for capturing surface dynamics. The ionic composition of soluble material in the Gobi Desert salt sample has been detailed in prior work,^34^ with the specific molar concentration percentages shown in Table 1. The salts were collected from harsh environments and conditions of high salinity, which are inhospitable to most organic life forms, resulting in a minimal organic presence within these salts. Additionally, carbonate minerals were identified in the mineral/salt mixtures,^34^ but due to low solubility they were not extracted and studied in this study.

**Table 1:** Ionic Composition of the Gobi Desert Salt Sample

ions	NO_3_^–^	SO_4_^2–^	Cl^–^	Ca^2+^	Mg^2+^	K^+^	NH_4_^+^	Na^+^
molar fraction	1%	38%	4%	7%	22%	1%	0%	27%

### 2.1 APXPS and NEXAFS Experiments

The APXPS and NEXAFS experiments were conducted at the X07DB *In Situ Spectroscopy* beamline of the Swiss Light Source (SLS) at the Paul Scherrer Institute (PSI), with the end station as described in previous literature.^35^ The preparation method of the salt sample from the Gobi Desert includes several steps. The sample was first dissolved in ultrapure water (Fluka TraceSelect Ultra; Water ACS reagent) and subsequently drop-cast onto a sample holder. The sample holder was then heated to 40 °C to expedite the evaporation and recrystallization of the salt. Following this, the sample was placed into the vacuum chamber maintained at a base pressure of 6 × 10^–8^ mbar. The salt layer formed is absent of large crystals and well mixed, which makes the XPS experiment less sensitive to the measured spot.

The chamber was kept at high vacuum conditions before and between measurements, with the pressure increased (up to 1 mbar) by introducing either argon (Ar) gas or water vapor (H_2_O) during the measurement. The RH was adjusted by lowering the temperature while maintaining constant pressure. The setup was calibrated before starting the experiments, precisely finding the saturation temperature as well as eventual offsets in the temperature reading. The RH was adjusted by lowering the temperature while maintaining constant pressure. Baratron capacitance manometers constantly measured the pressure inside the chamber, and we ensured it remained constant and below the saturation pressure of the set temperature. The temperature at the surface of the cryo sample holder was regulated by adjusting the flow of nitrogen gas, which passed through a coil submerged in liquid nitrogen outside the chamber system. The substrate temperature was measured a few millimeters from the coldest point in the center of the substrate using a thermocouple spot-welded to its side. It is important to note that the XPS analysis chamber itself was maintained at ambient temperature. The temperature difference between the sample surface and the thermocouple was routinely calibrated by determining the frost point based on water vapor partial pressure. The frost point was identified by a sudden drop in the water vapor partial pressure due to ice condensation. Typically, this temperature offset ranged from 1.0 to 1.5 K.

To reach a steady state, characterized by constant pressure and constant XPS signal intensity, the system was stabilized at each new RH condition for at least 10 min. At RH = 0%, Ar was added to prevent surface charging effects during measurements. At RH ≠ 0%, water vapor was introduced through a stainless-steel capillary (1.6 mm inner diameter), with varying conditions for each measured RH detailed in Table S1. The water used in the experiments was kept at a controlled temperature and degassed through three cycles of freezing, pumping, and thawing. Due to the extended duration of XPS and NEXAFS measurements relative to the limited available beamtime, the RH values were carefully selected to represent a broad range. Additionally, RH was kept below the deliquescence point to prevent artifacts, such as bubbling, that can occur during X-ray measurements on a partly deliquesced viscous surface.

The X07DB *In Situ Spectroscopy* beamline provides soft X-rays with energies from 300 to 1500 eV and with a resolving power >3000. The beam size at the sample surface was approximately 0.3 mm × 0.3 mm. Linearly polarized light was used throughout the experiments. Wide range scans (survey scans) of the Gobi Desert salt sample were acquired with a photon energy of 1000 eV (Figure S1). Photoemission spectra of O 1s, Na 2s, S 2p, Cl 2p, and Mg 2p orbitals were collected at three different photoelectron mean escape depths (MED) of 0.9, 1.0, and 1.4 nm (also called probing depth) corresponding to kinetic energies (KE) of 300, 400, and 600 eV, respectively. The corresponding calculated binding energy (BE) ranges were aligned to aliphatic carbon (C 1s at 284.8 eV). Gaussian functions were used to fit the measured photoemission spectra after subtraction of a constant background. Partial electron yield NEXAFS spectroscopy is highly sensitive to the local coordination environment surrounding an emitting atom. For this reason, sodium K-edge, magnesium K-edge and oxygen K-edge NEXAFS spectra were acquired to investigate water adsorption and alterations in the local chemical surroundings. For sodium and magnesium, the KLL Auger peaks were acquired while scanning the photon energy across the absorption threshold of the respective element (1068–1110 eV for sodium and 1296–1394 eV for magnesium). For oxygen, in order to avoid traveling photoemission peaks, a kinetic energy window (387–407 eV) on the tail of the KLL Auger was acquired while scanning the photon energy across the edge (525–560 eV).

Elemental ratios at three different probing depths between O, Na, S, Cl, and Mg elements are computed from the respective photoemission spectra O 1s, Na 2s, S 2p, Cl 2p, and Mg 2p. To establish elemental ratios, several factors such as the photoionization cross section (σ), inelastic mean free path (IMFP), photon flux (*I*_0_), and the transmission function of the analyzer (*T*_F_) are considered. When conducting XPS measurements, the kinetic energy for each element remains consistent across different probing depths, thereby mitigating the impacts of IMFP and *T*_F_. This means that only σ and *I*_0_ have to be considered to normalize peak areas and calculate elemental ratios with varying photoelectron MED.

### 2.2 Computational Parameters

Classical molecular dynamics simulations were employed to elucidate molecular structure changes of an Epsomite crystal surface exposed to rising RH. The present computational setup rigorously follows from our previous work, investigating water adsorption on the MgCl_2_·8H_2_O surface.^31^ The MgSO_4_·7H_2_O Epsomite crystal surface was created starting from the Fortes et al.^36^ crystallographic structure that can be downloaded here.^37^ The crystal structure with initial dimensions of 1.189, 1.190, and 0.67 nm in the *XYZ* dimensions, was then replicated, 1 × 2 × 4 in the three dimensions, resulting in 256 water molecules, 32 Mg^2+^ and 32 SO_4_^2–^. The simulation box was equilibrated for 100 ps at constant volume and temperature (NVT) MD using a 0.1 fs time step at 300 K. Finally, the *X*-dimension was enlarged to 8 nm, resulting in two vacuum/(100) crystal interfaces.

On one (100) crystal surface we placed 52 water molecules solvating 3 sulfate ions, 3 Mg^2+^, 3 Na^+^, and 3 Cl^–^, resulting in a system with less than one water sublayer adsorbed on the crystal surface. To avoid clashes, the water sublayer/crystal system was then relaxed, annealing the temperature from 0 to 300 K in 100 ps using a 0.2 fs simulation time step. Finally, we performed a 1 μs constant temperature simulation (NVT) MD starting from the equilibrated water layer/Epsomite crystal.

Here, classical MD simulations were performed using the TIP4P/2005^38^ water model in combination with Madrid-2019 force field (hereafter, M-FF). The M-FF adopts a down scaling approach for the ionic charges, which makes it possible to model polarizable effects in highly concentrated aqueous salt solutions,^39,40^ making this force field a reasonable choice for modeling adsorbed water mono- or (sub)layers rich in ionic solutes on the surface of ionic crystals. However, the M-FF was not designed for crystalline salts and the development of classical MD force field working simultaneously for solid and molten salts in contact with aqueous solution(s) remains a daunting task. Therefore, in our simulations we utilized the M-FF to describe all the interactions in our system, but (a) we froze the positions of Mg, S, and water oxygen in Epsomite, and (b) we ensured unscaled charges for Mg and sulfate ions into the crystal. The rational for this methodological choice is that, at the initial solvation stage (RH = 3% or below) the adsorbed water quantity is limited, preventing crystal deliquescence. Moreover, combining different force fields (e.g., one for the aqueous layer and one for the crystal substrate) is not (in general) good practice.

The GROMACS 2021 molecular dynamics package was employed to run all the MD simulations,^41^ using a leapfrog integrator scheme.^42^ The temperature was set to 300 K using a stochastic velocity rescale thermostat and a coupling time of 0.1 ps.^43^ The real-space Coulomb and van der Waals interactions were cut off at a distance of 0.9 nm, which is the cutoff used in the original TIP4*P*/2005 water. The particle mesh Ewald method^44^ with a relative tolerance of 10^–5^, fourth-order cubic interpolation, and a Fourier spacing parameter of 0.16 were adopted to evaluate the long-range component of the Coulomb and van der Waals interactions. The water molecule geometries were constrained using the SETTLE algorithm.^45^

The postprocessing analysis of the MD trajectories was performed using the PLUMED plug-in.^46^ The crystal/water surface vs water/air interface of the adsorbed ionic species were calculated tracking the perpendicular distances from a Willard-Chandler (WC) dividing surface,^47^ which follows the roughness of the crystal interface, defined by the Epsomite heavy atoms (i.e., O, S and Mg), to the center of mass of the adsorbed ionic species. The first 1 ns of MD trajectory were removed for equilibration purposes and uncorrelated frames every 20 ps were used for this analysis.

## 3 Results and Discussions

### 3.1 Sample Characterization

**Figure 1:**
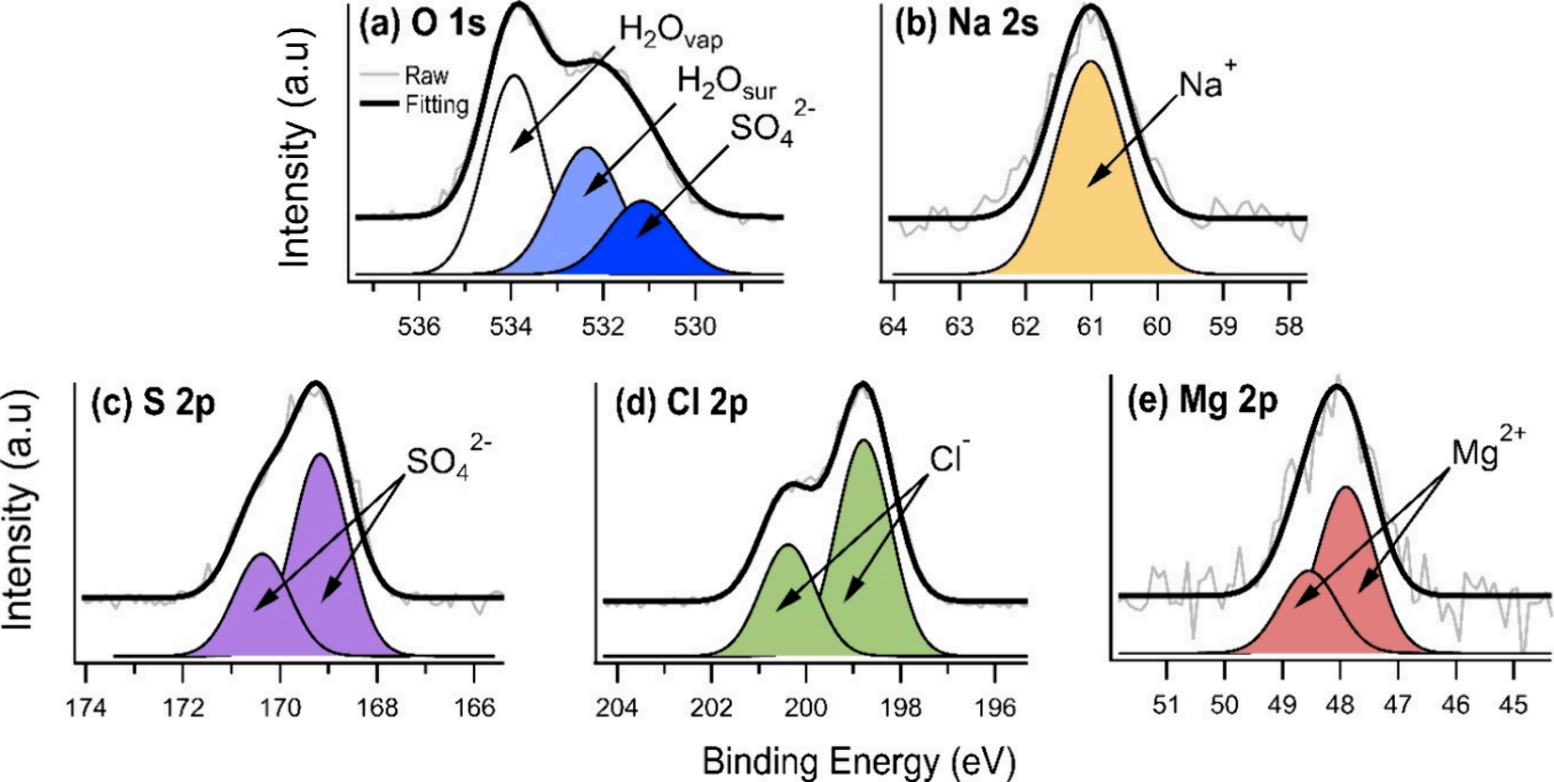
Photoemission spectra obtained with XPS for the Gobi Desert salt sample at 3% RH. The binding energies are aligned with aliphatic C 1s at 284.8 eV. The O 1s spectra were acquired with photon energy = 840 eV and kinetic energy = 300 eV. The other spectra (Na 2s, S 2p, Cl 2s, and Mg 2p) were acquired with various photon energies but with the same kinetic energy = 600 eV.

The Gobi Desert salt was analyzed using XPS, and the photoemission spectra for O 1s, Cl 2p, S 2p, Na 2s, and Mg 2p orbitals at 3% RH are depicted in Figure 1 (see Supporting Information for additional RH and KE data). In Figure 1a, the O 1s spectrum is deconvoluted into three components, representing sulfate (SO_4_^2–^), surface water (H_2_O_sur_), and water vapor (H_2_O_vap_), at BE of 531.15, 532.25, and 533.93 eV, respectively. The energy separation between SO_4_^2–^ and H_2_O_sur_ is approximately 1.20 eV, and between SO_4_^2–^ and H_2_O_vap_ it is approximately 2.80 eV, which align with a prior XPS study on a natural sample.^33^ Further, the Na 2s BE in Figure 1b is 61.01 eV, with a single sodium cation species (Na^+^) identified. In Figure 1c, the S 2p is associated with sulfate, with S 2p_3/2_ BE of 169.17 eV, while in Figure 1d, the Cl 2p corresponds to chloride (Cl^–^), with Cl 2p_3/2_ BE of 198.68 eV, and in Figure 1e, the Mg 2p corresponds to magnesium cation (Mg^2+^), with Mg 2p_3/2_ BE of 47.90 eV. The spin–orbit splitting values for S 2p, Cl 2p, and Mg 2p are 1.20, 1.60, and 0.64 eV, respectively.

### 3.2 Elemental Ratios with Varying Probing Depth

**Figure 2:**
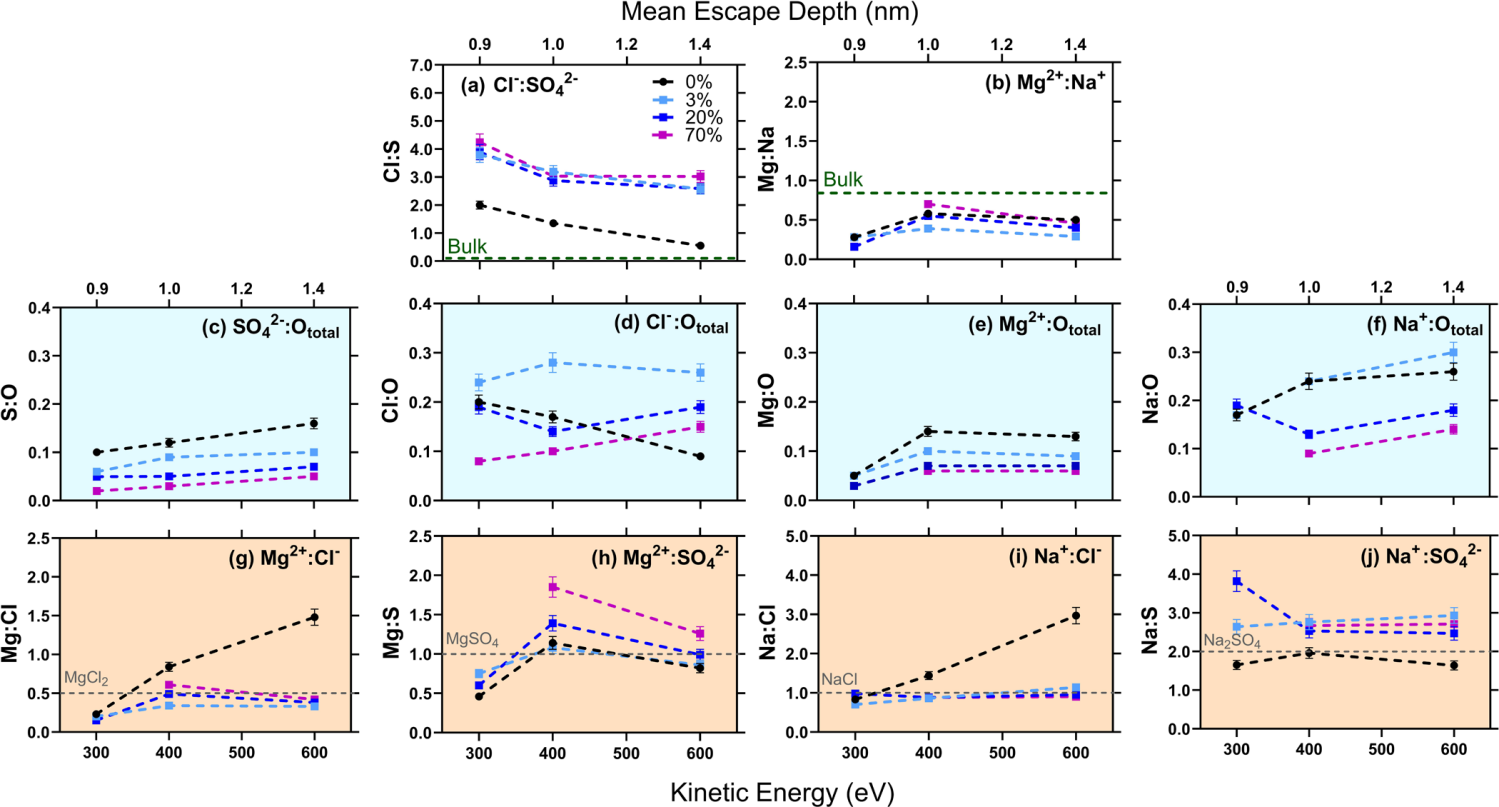
Elemental ratios with varying MED. The elemental ratios were acquired at 0%, 3%, 20%, and 70% RH. The *y*-axis shows the elemental ratios where the photoemission spectra S 2p, Cl 2p, Mg 2p, O 1s, and Na 2s are utilized for the respective elements S, Cl, Mg, O, and Na. The KE is shown on the bottom *x*-axes, and the corresponding MED is displayed on the top *x*-axes. From the elemental ratios, molar ratios between (a) anionic species, (b) cationic species, (c–f) anionic or cationic species to O_total_, and (g–j) anionic and cationic species can be established. The contribution of H_2_O_vap_ species is removed of the O element signal and O_total_ represents the cumulative signal of H_2_O_sur_ + SO_4_^2–^ species on the surface. The ionic ratios in bulk (measured by IC) are marked as green dashed lines in (a) and (b), and the elemental ratio of pure salts (MgCl_2_, MgSO_4_, NaCl, and Na_2_SO_4_) are marked in (g)–(j).

The molecular composition and the arrangement of species can be obtained by establishing elemental ratios at different probing depth (or photoelectron MED). The elemental ratios between O, Na, S, Cl, and Mg elements are calculated from the photoemission spectra (see Material and Methods). From the elemental ratios, the molar ratios between different species can be determined. As depicted in Figure 2, the elemental ratios (*y*-axis) were measured at 3 distinct photoelectron MED and 4 RH levels: 0%, 3%, 20%, and 70%. The contribution of gas-phase H_2_O_vap_ in O 1s spectra (Figure 1a) is removed in the O element signal to ensure that only surface species are considered in the analysis. As two distinct maxima are not observed on the O 1s photoemission spectra for the deconvoluted H_2_O_sur_ and SO_4_^2–^ species (Figure 1a), they are grouped together (H_2_O_sur_ and SO_4_^2–^) in the molar ratios as O_total_ to minimize errors.

Figure 2a showcases the variance in the molar ratio between two principal anionic species, sulfate and chloride, as RH increases. A notable shift in the Cl:S ratio is observed as RH transitions from 0% to higher values. At 0% RH, the Cl:S ratio decreases with MED, ranging from 2.0 to 0.5, indicative of a thin superficial layer that is rich in chloride. Note that the bulk ratio of Cl:S is around 0.1 (Table 1), so the surface is already Cl-enriched at 0% RH. Intriguingly, with a small RH increase to 3%, the Cl:S ratio increases to approximately 4.0 at the uppermost MED and to 3.0 at the two others. This implies a rather uniform distribution of chloride throughout the XPS probing depth investigated in the work. Additionally, no further changes in Cl:S ratio were noticed when RH increases to 20% and then to 70%. Figure 2b shows that the molar ratio between the most abundant cationic species (Mg^2+^ and Na^+^) is enhanced from the second MED, suggesting that the topmost surface layer is depleted of Mg^2+^ in comparison of Na^+^. Thus, the change of RH mostly affects the molar ratio between anions, i.e., Cl^–^ is relatively enriched compared to SO_4_^2–^.

Figure 2c displays a decreasing trend in the S:O ratio as RH increases from 0% to 70%, which is attributed to water adsorption on the surface that gradually solvates sulfate anions. Moreover, the parallel evolution of the S:O ratio with the probing depth at different RH values remains constant, suggesting a constant distribution of sulfur- and oxygen-containing species. The profiles are also consistent with the existence of surface water even at 0% RH, also given the observation that the S:O ratio consistently falls below the benchmark value of 0.25 expected for pure sulfate. Furthermore, surface water evidence even at 0% RH is seen in the O 1s XPS spectra in the Supporting Information.

The Cl:O ratio depicted in Figure 2d is not monotonic with increasing RH. When RH = 0%, the Cl:O ratio decreases as the MED increases, mirroring the Cl:S ratio trend, given the association of sulfur and oxygen with sulfate. At 3% RH, the Cl:O ratio noticeably increases and maintains a consistent value around 0.25 for the full range MED. This observation indicates a significant surge in chloride ions following water adsorption. Conversely, at 20% and 70% RH, the Cl:O ratio decreases compared to its value at RH = 3%, especially at the surface. This change is likely attributed to the dilution with water.

The Mg:O and the Na:O ratios can be considered simultaneously. In Figure 2e, as the RH increases from 0% to 70%, the Mg:O ratio declines, suggesting gradual solvation of magnesium in water. In contrast, the Na:O ratio in Figure 2f follows a similar pattern as that of the Cl:O (Figure 2d) ratio, indicating a correlation between the behavior of Na^+^ and Cl^–^ ions.

Figure 2g–j display the cation/anion ratios and include the standard ratios for pure salts (MgCl_2_, MgSO_4_, NaCl, and Na_2_SO_4_) as dashed gray lines as guides for the reader. In general, ratios approximating the dashed lines might imply a compositional similarity to a designated reference salt within the sample, although this correlation is not deterministic. At 0% RH, the Mg:Cl ratio depicted in Figure 2g exhibits an increase with MED, diverging from the benchmark value for MgCl_2_. This reflects the steep increase in the Cl:S ratio observed at 0% RH in Figure 2a, indicating a lack of correlation between chloride and either magnesium or sulfate at higher MED levels. Contrastingly, at 3%, 20%, and 70% RH, the Mg:Cl ratios dip below the theoretical value for pure MgCl_2_. It is noteworthy that the Mg:Cl and Cl:S elemental ratios exhibit similarities, hinting at a potential association between Mg^2+^ and SO_4_^2–^. This potential correlation is further explored in Figure 2h, where the Mg:S ratios at 0%, 3%, and 20% RH align closely with the reference value for MgSO_4_. However, at 70% RH, the Mg:S ratio is increased, a phenomenon that will be discussed later.

Figure 2i displays the Na:Cl ratio. Initially, at the uppermost MED of 0.9 nm, the ratios at all four RH levels align closely with the value for pure NaCl. At increased MED, the 0% RH case exhibits an increasing trend, indicative of Cl^–^ depletion compared to Na^+^. It is therefore possible that a portion of the Na^+^ is paired with other counterions, such as SO_4_^2–^, as also suggested by the Mg:S ratio in Figure 2h. Conversely, at the other three nonzero RH levels, across all MED levels, the Na:Cl ratio remains consistent with the value expected for pure NaCl, supporting the likelihood of sodium and chloride combining to form the salt crystal NaCl.

In Figure 2j, the Na:S ratio at 0% RH maintains a consistent trend across the MED levels, aligning closely with the benchmark value for pure Na_2_SO_4_ salt. In contrast, at the other nonzero RH levels, the Na:S ratios are greater than the reference value for Na_2_SO_4_. Taking into account the Na:Cl ratios illustrated in Figure 2i, it is reasonable to assume that at 0% RH, the Na^+^ predominantly associates with SO_4_^2–^, whereas at elevated RH levels, the Na^+^ preferentially binds to Cl^–^ to form NaCl. Therefore, at these relative humidity levels, the Na:S ratio indicates the proportions of distinct Na^+^ and SO_4_^2–^ containing species.

Based on the elemental ratios depicted in Figure 2, a plausible scenario is the presence of a thin topmost layer of pure NaCl resting above a blend of Na_2_SO_4_ and MgSO_4_ at 0% RH. As RH increases, this NaCl layer thickens. In this configuration, chloride ions are paired with sodium ions, while sulfate ions are linked with magnesium ions. To validate this hypothesis derived from the elemental ratios, NEXAFS analysis can be employed as a robust tool to provide evidence of the crystalline structures, thereby confirming which species exist.

### 3.3 Surface Composition by NEXAFS

**Figure 3:**
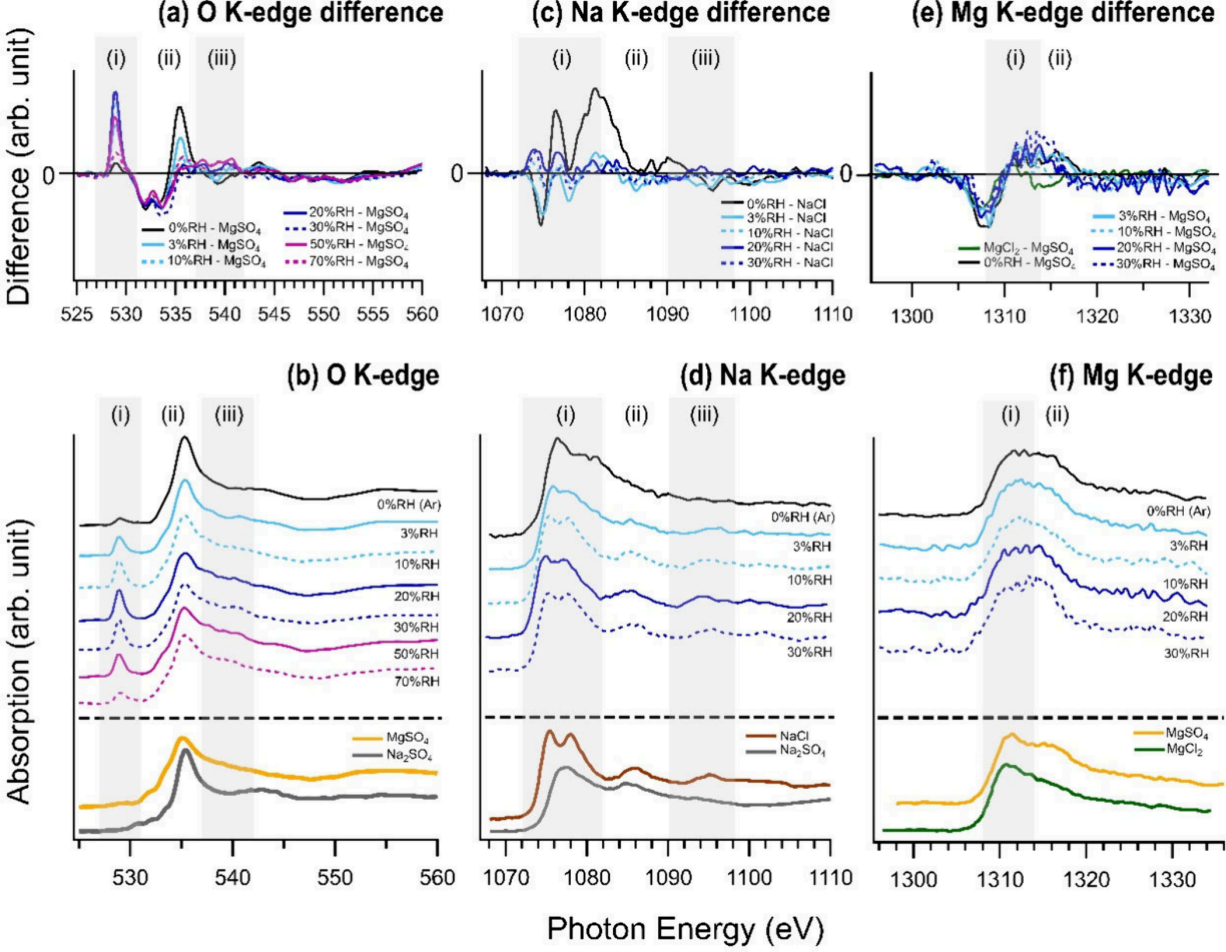
NEXAFS spectra for the Gobi Desert salt. The spectra were acquired at RH between 0 and 70% for (b) oxygen K-edge, (d) sodium K-edge, and (f) magnesium K-edge. (a), (c), and (e) are the difference spectra between the Gobi Desert salt spectra and the reference spectra, including (a) oxygen K-edge with the MgSO_4_ at 0% RH as the reference, (c) sodium K-edge with NaCl at 0% RH as the reference, and (e) magnesium K-edge with MgCl_2_ at 0% RH as the reference. Normalization was done prior to subtraction.

Surface alterations due to changing RH are evident in the NEXAFS spectra presented in Figure 3. This analysis encompasses three spectral edges: the oxygen K-edge, sodium K-edge, and magnesium K-edge. The difference spectra are calculated and included in the figure with MgSO_4_ taken as a reference for O K-edge and Mg K-edge, and NaCl for Na K-edge. In the oxygen K-edge spectra (Figure 3a,b), three distinct regions can be identified: (i) 527–531 eV, (ii) 533–538 eV, and (iii) 538–542 eV. The feature in region (i) is probably associated with the presence of metal oxides,^48^ which could be found in the salts of the Gobi Desert. The distinct peak shape observed in region (ii) for dry conditions is similar to that found in ammonium sulfate,^49^ magnesium sulfate,^33^ and sodium sulfate,^31^ suggesting that it is associated with the oxygen atoms in sulfate ions. The feature in region (iii) is associated with adsorbed water.

At 0% RH, the O K-edge spectrum (Figure 3b) displays a peak intensity in region (ii) that closely resembles the pure reference Na_2_SO_4_ spectra, indicating a dominant presence of sodium sulfate at dry conditions, matching the above hypothesis based on elemental ratios derived by XPS. As RH increases, the spectrum lines begin to flatten, taking on characteristics more akin to MgSO_4_ (Figure 3b). However, water adsorption can also contribute to the flattening of the spectrum lines. To facilitate comparison, difference spectra were plotted in Figure 3a, with the MgSO_4_ spectrum utilized as the baseline for subtraction. This demonstrates that the disparity between the desert salt and MgSO_4_ declines as RH rises, particularly in region (ii) which is independent from water adsorption. MgSO_4_ has a propensity to bind with water, forming various hydrates, and a higher water content can potentially result in a more diffuse spectrum.

In the sodium K-edge spectra shown in Figure 3d, three regions are identified: (i) 1072–1082 eV, (ii) 1082–1090 eV, and (iii) 1090–1098 eV. Two reference sodium K-edge spectra, for NaCl and Na_2_SO_4_, are included for comparison. These spectra were derived from pure salt samples previously analyzed.^31^ In region (i), NaCl exhibits two peaks, whereas Na_2_SO_4_ displays only one. Region (ii) is similar for both salts, while region (iii) features a minor peak for NaCl but none for Na_2_SO_4_. Regarding the Gobi Desert salt at 0% RH, the spectrum diverges from that of both NaCl and Na_2_SO_4_, suggesting that Na^+^ ions are bound with both counterions across the surface at 0% RH throughout the depth analyzed by NEXAFS. As RH increases, the spectra progressively shift to a more NaCl-like appearance. This transformation is clearly depicted in both the difference spectra shown in Figure 3c and the original spectra in Figure 3d. This is in fair agreement with the elemental ratios discussed previously.

In Figure 3f, the magnesium K-edge spectra are displayed with MgCl_2_ and MgSO_4_ serving as reference comparisons. There are two discernible regions within the magnesium spectra: (i) 1308–1313 and (ii) 1313–1318 eV, identified based on the characteristic features of MgCl_2_ and MgSO_4_ spectra. In region (i), MgCl_2_ and MgSO_4_ exhibit similarities, while region (ii) is marked by a peak exclusive to MgSO_4_. When examining the desert salt spectra, a peak-like feature emerges in region (ii), implying a definitive association between Mg^2+^ and SO_4_^2–^. While the spectrum shapes alone do not entirely preclude the possibility of MgCl_2_ being present, the observed 1:1 Na:Cl ratio clearly indicates that most chloride ions are paired with sodium ions, rather than with magnesium ions.

### 3.4 Molecular Dynamics Results

**Figure 4:**
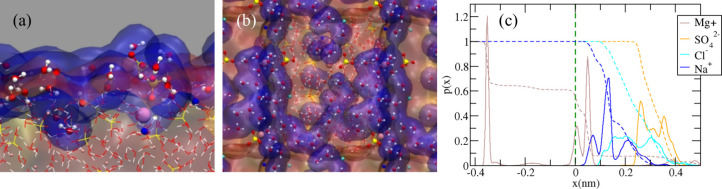
Panel (a) side view of the final snapshot after 1 s MD simulation. The blue surface spotlights the adsorbed water submonolayer on the epsomite crystal surface. Atom color code: Mg (pink), S (yellow), O (red), H (white), Cl (cyan), and Na (blue). The large Mg atom is adsorbed Mg^2+^ on the crystal surface. Panel (b) is the top view of panel (a). (c) Probability density profiles for individual species (not normalized) as a function of the *x*-coordinate perpendicular to the crystal/water Willard–Chandler interface (*x* = 0). Dashed lines correspond to the cumulative distribution from the gas (*x* = 0.4 nm) toward the crystal phase.

To elucidate the molecular mechanism(s) underlying the crystal/water vs water/air surface preference of the adsorbed ionic species accompanying increasing RH, MD simulations were conducted. Gobi Desert salt is a combination of different salt hydrate crystalline structures. Given the constraints related to the scale of atomistic modeling, epsomite (MgSO_4_·7H_2_O) crystal is used as a representative model for crystals found in the Gobi Desert salt in our MD simulations. This choice is grounded in the prevalence of epsomite as a commonly occurring hydrated structure in deserts and salt lakes.^50^ The epsomite, (MgSO_4_·7H_2_O) crystal was created with its surface coated by a submonolayer of water (Figure 4a,b), interspersed with trace amounts of Na^+^, Mg^2+^, Cl^–^, and SO_4_^2–^ ions. It is a daunting task to do a rigorous quantitative match between the amount of adsorbed water molecules in the simulation system and the experimental RH. Nevertheless, it is reasonable to assume that the modeling of an adsorbed water submonolayer on the crystal epsomite surface corresponds to a low RH experimental scenario.

At the simulated surface (Figure 4c), we report the diffusion and solvation of one Mg^2+^ in an interstitial crystal region (i.e., peak at *z* = −0.4 nm). Chloride ions gravitate toward the liquid–solid interface, whereas sulfate ions tend to remain closer to the liquid–air interface: this conclusion aligns with our prior study on the surface of MgCl_2_·8H_2_O.^33^ Notably, this finding contrasts with our experimental observations, where chloride is observed to rapidly accumulate on the surface as RH increases. This discrepancy implies that the swift enrichment of Cl^–^ on the surface is instigated by mechanisms other than the ion-selective surface propensity within a homogeneous mixture layer. Moreover, crystal solvation and nucleation processes at the epsomite surface as a function of the RH may play a role in the Cl^–^ enrichment at the air/liquid water surface, as discussed below. However, these processes are not easy to capture due to the limitations of classical MD and the lack of a stringent and quantitative matching between the experimental RH and the amount of adsorbed water.

### 3.5 Mechanism of Water-Driven Surface Transformation

The elemental ratios depicted in Figure 2 demonstrate that additional surface water begins to adsorb at nonzero RH, but is accompanied by the formation of NaCl, seen in both the Na:Cl ratio in Figure 2i and the NEXAFS spectra in Figure 3. At 0% RH, the Na^+^:SO_4_^2–^ molar ratio (Figure 2j), the O K-edge spectra (Figure 3a,b), and the Na K-edge spectra (Figure 3c,d) collectively indicate an association between sodium and sulfate, likely forming Na_2_SO_4_. As RH increases, the O K-edge spectra (Figure 3a,b), Na K-edge spectra (Figure 3c,d), and the Mg K-edge spectra (Figure 3e,f) collectively demonstrate a shift in association from sodium to magnesium for sulfate ions. The transition appears connected to the formation of NaCl on the uppermost surface layer, associated with the adsorption of water on the surface even at RH levels as low as 3%.

It is essential to consider the bulk ionic molar fractions of the salt, as outlined in Table 1. The data shows that sulfate ions comprise the primary anion at 38%, with sodium and magnesium ions serving as the principal cations at 27% and 22%, respectively. In contrast, chloride ions constitute a relatively minor 4% of the total ionic content, making them almost ten times less concentrated than sulfate ions. Nevertheless, the chloride ions appear remarkably mobile and migrate efficiently to the surface, where the NEXAFS indicates that they combine with Na^+^ to form NaCl that appears to homogeneously cover the surface of the Gobi Desert salt. This phenomenon illustrates how a relatively minor bulk component can dominate the surface. It is also noteworthy that while NaCl typically functions as the core of effloresced sea salt particles,^51,52^ but in the case of desert salt, the core consists of MgSO_4_, with NaCl adopting the role of a coating material. This occurrence is likely linked to the dominant sulfate ionic composition, though caution should be noted regarding the sample preparation process, where the brine was heated to 40 °C to evaporate the water. Nevertheless, the behavior of the sulfate-dominating natural brine highlights the importance of considering microenvironmental factors, such as diurnal RH cycles, in understanding the geochemical processes occurring in arid regions like the Gobi Desert.

**Figure 5:**
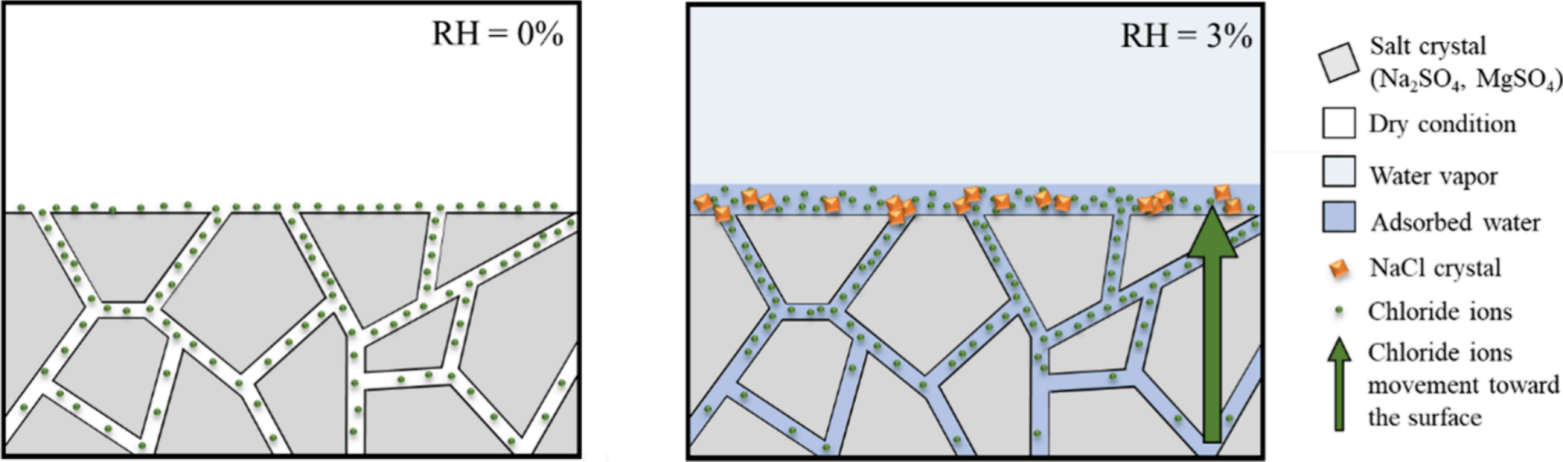
Gobi Desert salt grain boundaries. Chloride ion enhancement at the surface, with formation of NaCl crystal upon water adsorption.

Figure 5 illustrates a hypothesized mechanism for how the salt–gas interface transforms on the Gobi Desert salt. At 0% RH, NaCl or Na_2_SO_4_ crystals were not present as pure crystals, as indicated by the Na K-edge NEXAFS spectrum in Figure 3d, which differs significantly from that of pure NaCl or Na_2_SO_4,_. This suggests that the chloride ions were not part of crystalline structures but likely existed in a glassy state between the crystals (grain boundaries). These glassy materials are more susceptible to absorbing water molecules from the gas phase than crystalline structures, and as RH increases, their increased fluidity allows ion movement.

Additionally, the other crystals, including MgSO_4_, hydrate by absorbing water vapor even at low RH levels. This hydration causes volumetric expansion of the crystals, which in turn squeezes the grain boundaries and pushes the ions within toward the surface. This could serve to preferentially segregate certain species, and likely in this case drive Na^+^ and Cl^–^ ions toward the surface. The expelled Na and Cl ions meet at the surface and form NaCl, where the RH remains well below the efflorescence relative humidity (ERH) of NaCl (∼45% RH).

The proposed mechanism is consistent with the notion of surface hydration layers affecting the mobility of ions in crystalline salts. The presence of water, even in small amounts, significantly alters the surface energetics and chemistry of hygroscopic materials. It is also worth noting that the crystalline structure, specifically the presence and arrangement of grain boundaries and different domains, plays a crucial role in this process. Further experimental and theoretical work, particularly *in situ* spectroscopic studies and advanced computational modeling, could provide deeper insights into the kinetics and thermodynamics of such surface transformations. Moreover, expanding this experimental work to lakebed samples from various arid regions with differing ionic compositions could enhance our understanding of these processes and their global prevalence. This can help inform future geochemical and mineralogical research as well as have broader applications in environmental science and materials engineering.

## 4 Conclusions

This study presents a comprehensive analysis of surface transformation of Gobi Desert salts under changing RH levels and reveals intricate mechanisms governing the surface composition. Elemental ratios, combined with NEXAFS and APXPS findings, indicate the presence of a NaCl/Na_2_SO_4_ mix on the salt surface under dry conditions, transitioning to predominantly NaCl with increasing RH. The migration of chloride ions to the surface, despite their relatively minor presence in the bulk salt, highlights the nuanced interactions that influence the behavior of desert salt surfaces. MD simulations further enriched our understanding, albeit presenting discrepancies with experimental observations. This study underscores the significant role of water adsorption in driving surface transformations, even at minimal RH levels. Recognizing the behaviors of desert salts, particularly in the light of their potential atmospheric and ecological implications, remains paramount for our deeper comprehension of Earth’s arid regions and their broader impacts.

## Supporting Information Available

Figure S1: Wide range spectra of Gobi Desert salt sample at different RH levels. Figure S2: Images of the desert salt sample at 0% and 3% RH. Figures S3–S8: Peak fitting for C 1s, Cl 2p, O 1s, S 2p, Mg 2p, and Na 2s at varying RH levels. Table S1: Calculation of RH from the water equilibrium pressure at corresponding temperatures. Table S2: Bulk composition ratios between main ions based on molar fractions. Tables S3–S7: Deconvolution of XPS photoemission spectra for Cl 2p, Mg 2p, S 2p, Na 2s, and O 1s (PDF)
