# Supplementary material for: Formation of Sodium Chloride on the Surface of Sulfate-Rich Gobi Desert Salt in Response to Water Adsorption
Source: ACS EST Air. 2024 Oct 17;1(11):1373–82. doi: 10.1021/acsestair.4c00092 (PMC11555638; doi:10.1021/acsestair.4c00092)
Supplement: Supplementary file 1 — ea4c00092_si_001.pdf [file ea4c00092_si_001.pdf]

Supporting Information

for

Formation of Sodium Chloride on the Surface of Sulfate-Rich Gobi Desert Salt in Response to Water Adsorption

Nicolas Fauré[1], Jie Chen[2], Luca Artiglia[3], Markus Ammann[3], Thorsten Bartels-Rausch[3], Zamin A. Kanji[2], Sen Wang[4], Jan B. C. Pettersson[1], Erik S. Thomson[1*], Ivan Gladich[5,6*], and Xiangrui Kong[1*]

[1]Department of Chemistry and Molecular Biology, Atmospheric Science, University of Gothenburg, SE-41390 Gothenburg, Sweden
[2]Department of Environmental Systems Science, ETH Zürich, Zürich, 8092, Switzerland
[3]Laboratory of Atmospheric Chemistry, Paul Scherrer Institute, CH-5232 Villigen PSI, Switzerland
[4]Shaanxi Key Laboratory of Earth Surface System and Environmental Carrying Capacity, Northwest University, Xi'an 710127, China
[5]European Centre for Living Technology (ECLT), Dorsoduro, Calle Crosera, 30124 Venice, Italy
[6]Qatar Environment and Energy Research Institute, Hamad Bin Khalifa University, P.O. Box 31110, Doha, Qatar

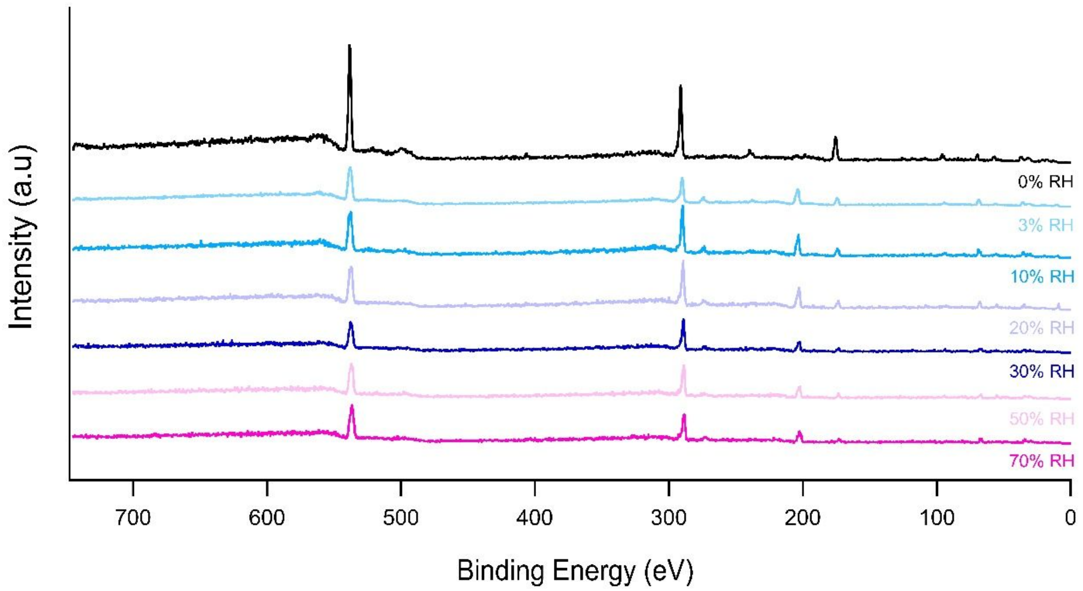

Figure S1: Wide range spectra (survey) of the desert salt sample at different RH. The surveys were acquired at a photoenergy of 1000 eV.

Table S1: Calculation of RH from the water equilibrium pressure $P_{equilibrium}$ at the corresponding temperature T and the actual water vapor pressure $P_{measured}$ that was dosed and measured in the APXPS in-situ cell.

RH (%)	T (°C)	Pequilibrium (mbar)	Pmeasured (mbar)	RH in Figures (%)
2.9-3.2	25	31.67	0.92-1.02	3
9.6	6	9.35	0.9	10
19.4	-4	4.55	0.88	20
30.4	-10	2.08	0.87	30
50.3	-16	1.73	0.87	50
68.6	-20	1.25	0.86	70

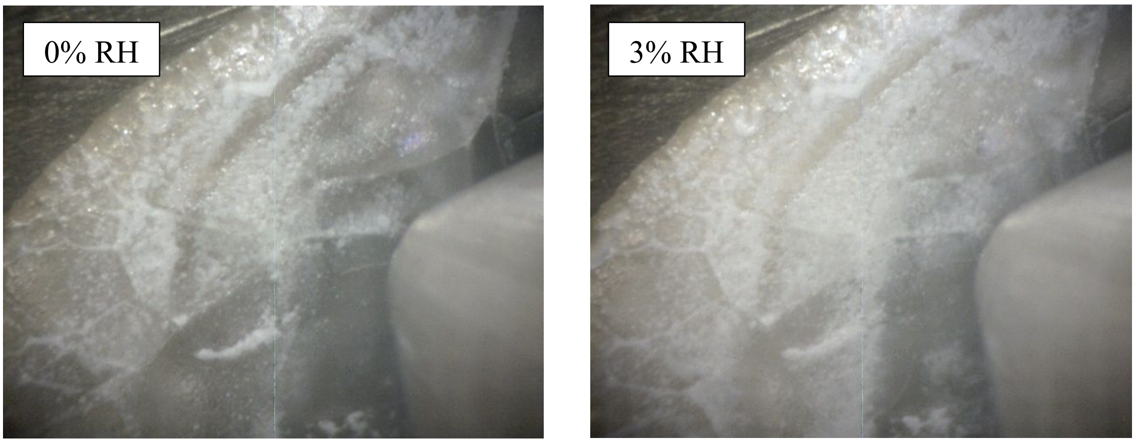

Figure S2: Images of the desert sample salt at 0% and 3% RH taken in the APXPS in-situ cell prior to measurements.

Table S2: Bulk composition ratios between the main ions based on the molar fractions obtained by IC. Ratios were obtained by dividing the molar fraction values of the ions in the raw (x) by the ones in the column (y).

	**Na^+**	**Mg^{2+}**	**SO_4^{2-}**	**Cl^-**
**Na^+**	1.00	0.64	1.00	0.10
**Mg^{2+}**	1.56	1.00	1.56	0.17
**SO_4^{2-}**	1.00	0.64	1.00	0.10
**Cl^-**	9.75	6.25	9.75	1.00

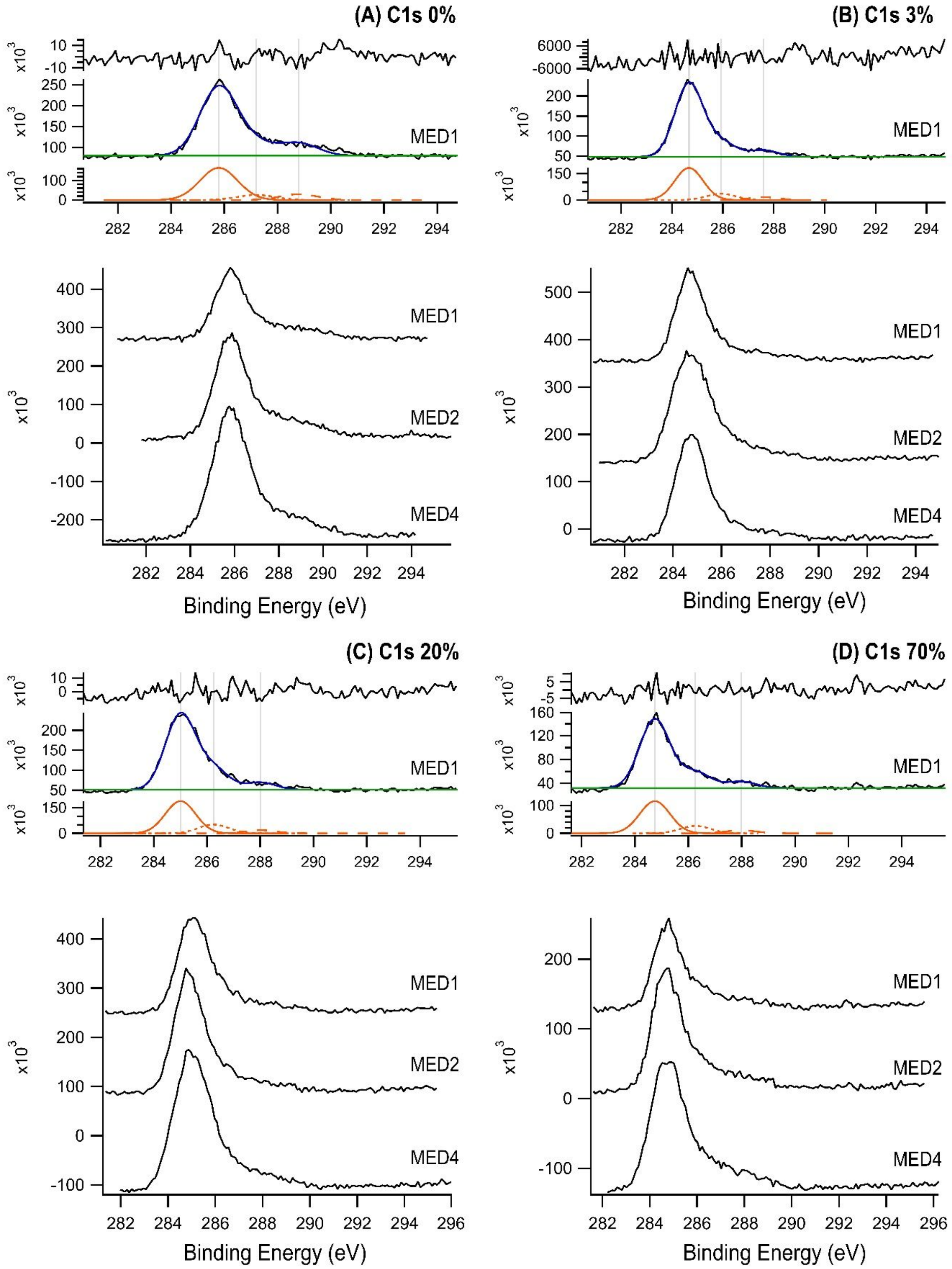

Figure S3: C 1s peak fitting of desert salt sample for (A) C 1s 0%RH, (B) C 1s 3%RH, (C) C 1s 20%RH, (D) C 1s 70%RH, MED1, 2, 4 refers to the Mean Escape Depth (Figure 2) of 0.9, 1.0 and 1.4 respectively.

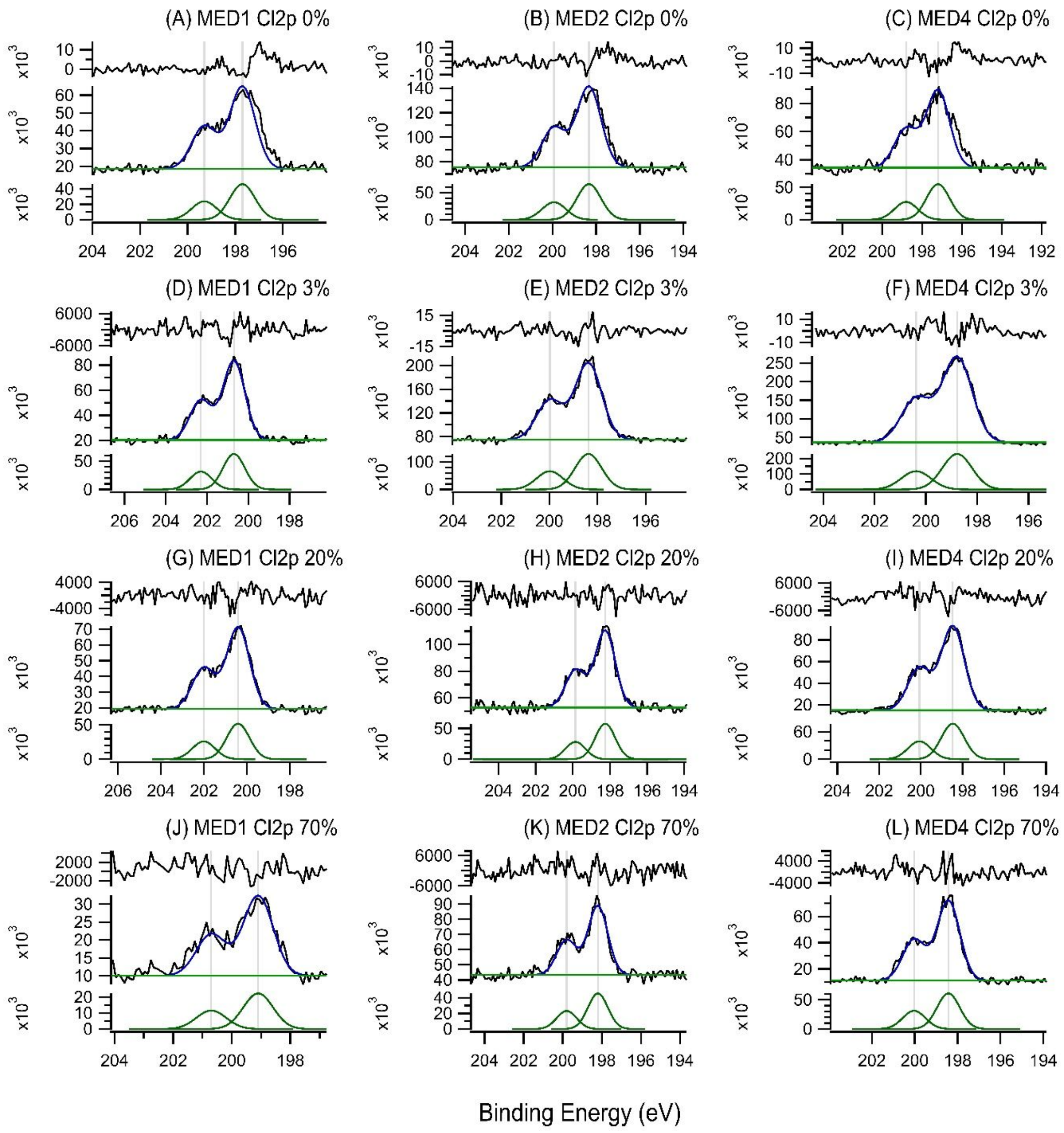

Figure S4: Cl 2p peak fitting of desert salt sample. (A), (B), (C), are 0% RH, (D), (E), (F) are 3% RH, (G), (H), (I) are 20% RH and (J), (K), (L) are 70% RH. MED1, 2, 4 refers to the Mean Escape Depth (Figure 2) of 0.9, 1.0 and 1.4 respectively.

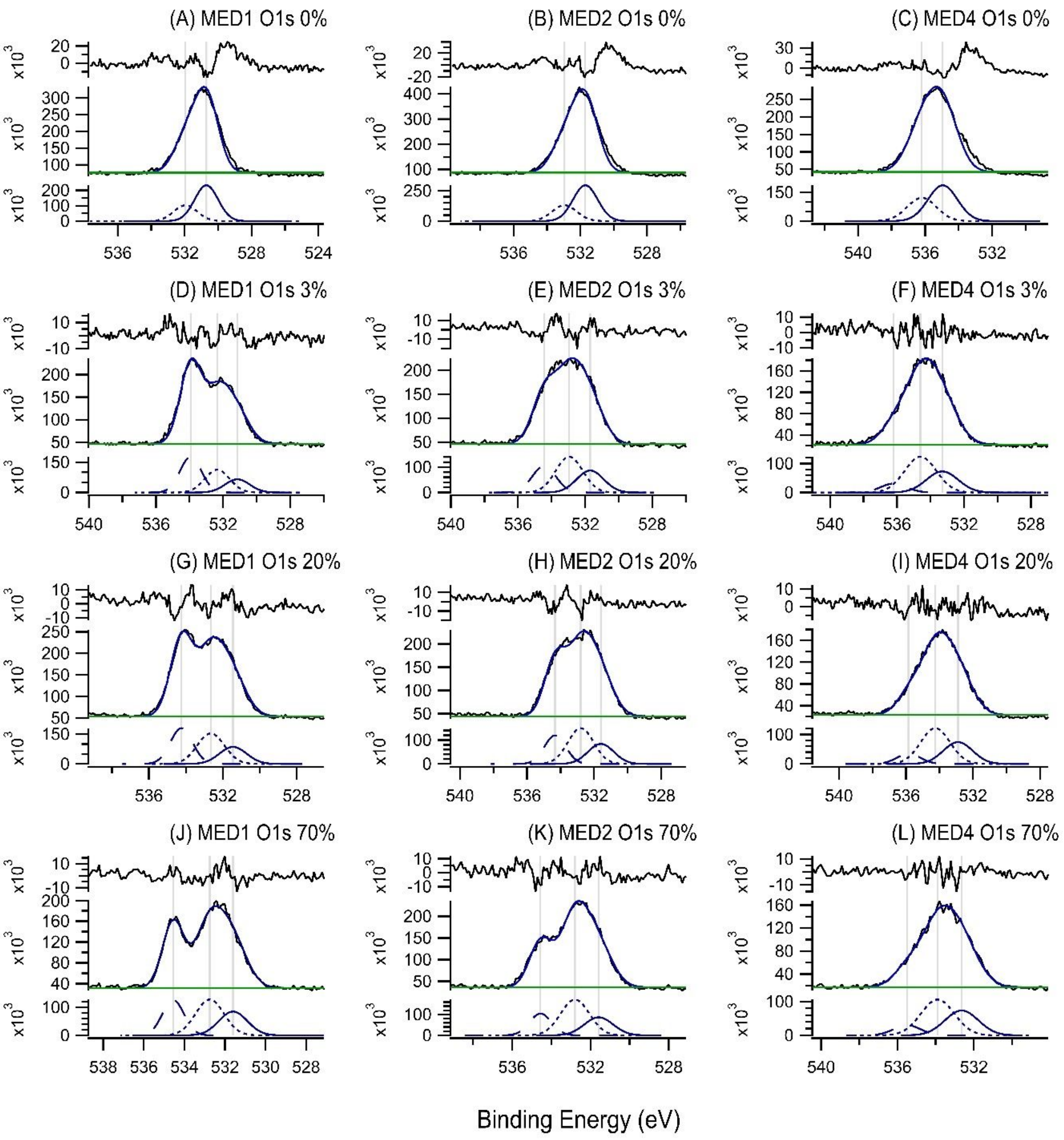

Figure S5: O 1s peak fitting of desert salt sample. (A), (B), (C), are 0% RH, (D), (E), (F) are 3% RH, (G), (H), (I) are 20% RH and (J), (K), (L) are 70% RH. MED1, 2, 4 refers to the Mean Escape Depth (Figure 2) of 0.9, 1.0 and 1.4 respectively.

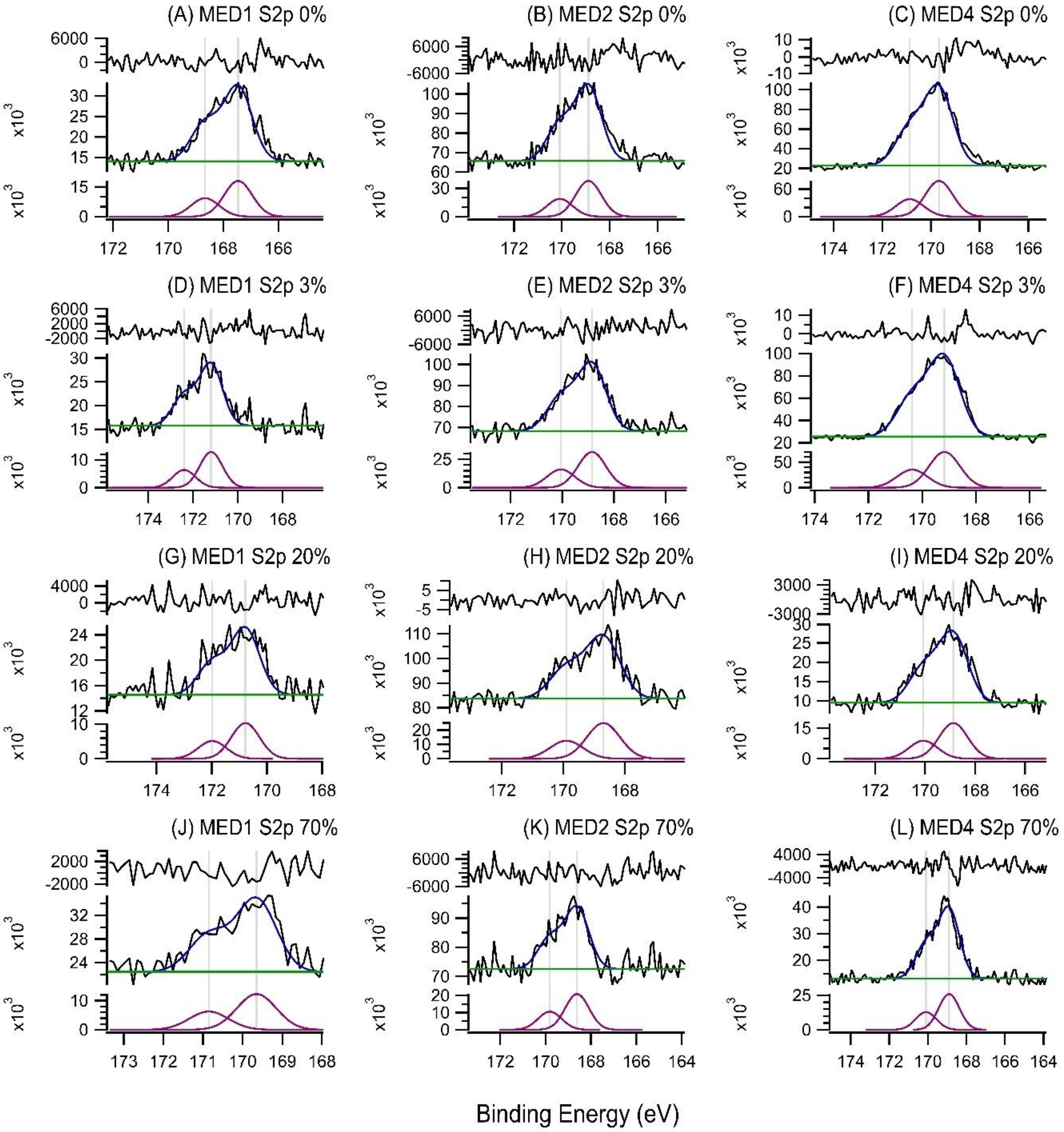

Figure S6: S 2p peak fitting of desert salt sample. (A), (B), (C), are 0% RH, (D), (E), (F) are 3% RH, (G), (H), (I) are 20% RH and (J), (K), (L) are 70% RH. MED1, 2, 4 refers to the Mean Escape Depth (Figure 2) of 0.9, 1.0 and 1.4 respectively.

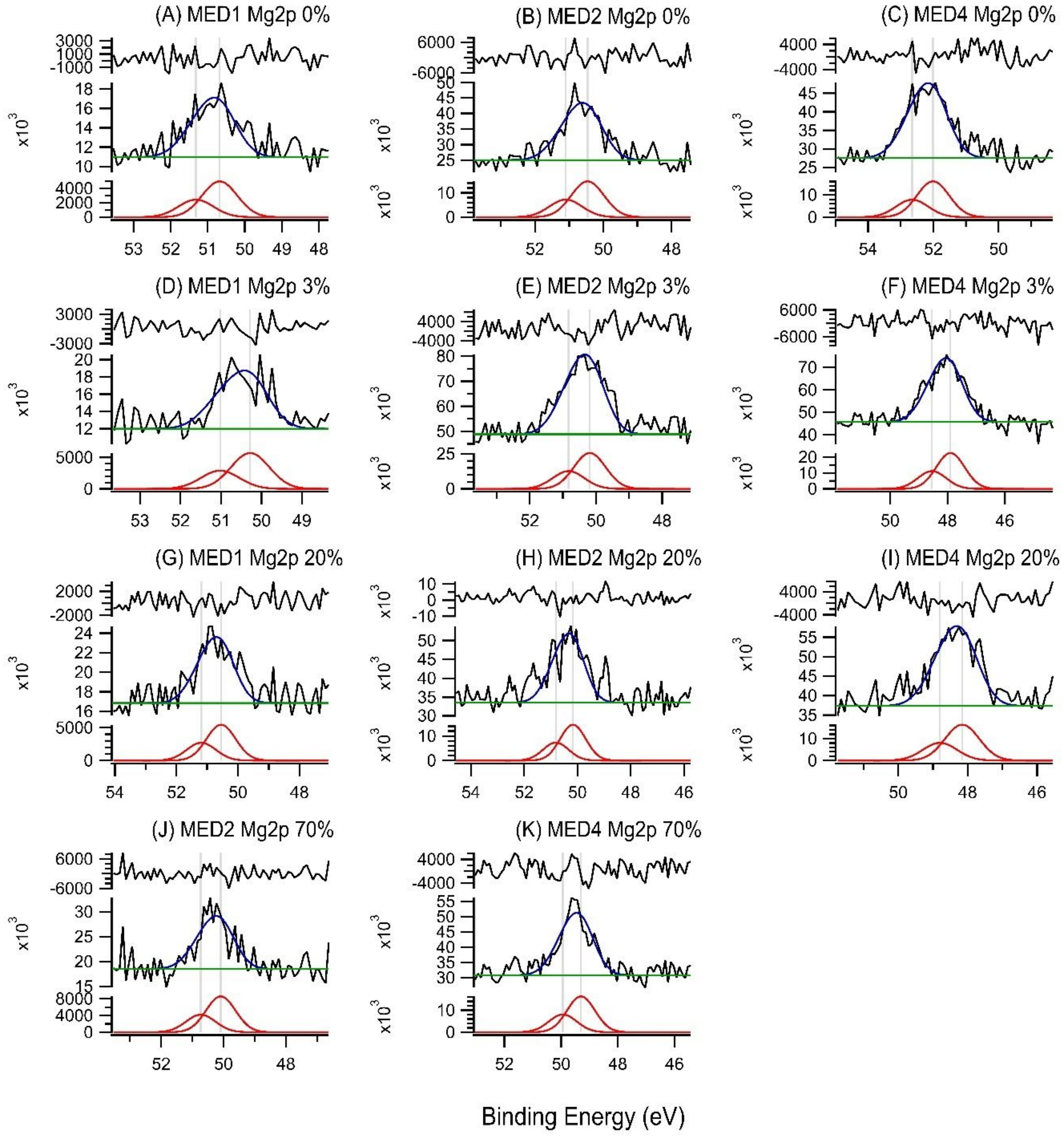

Figure S7: Mg 2p peak fitting of desert salt sample. (A), (B), (C), are 0% RH, (D), (E), (F) are 3% RH, (G), (H), (I) are 20% RH and (J), (K) are 70% RH. MED1, 2, 4 refers to the Mean Escape Depth (Figure 2) of 0.9, 1.0 and 1.4 respectively.

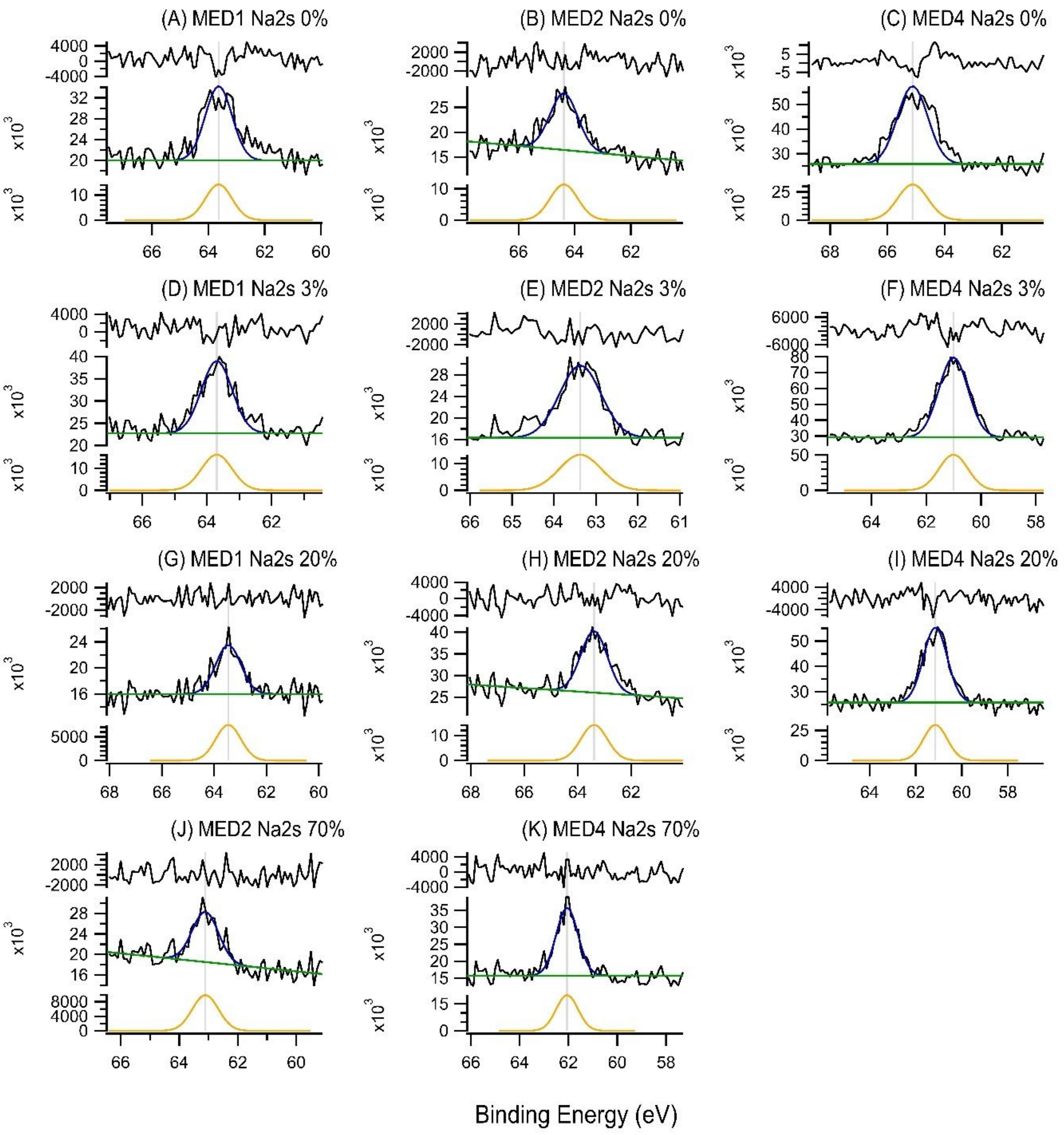

Figure S8: Na 2s peak fitting of desert salt sample. (A), (B), (C), are 0% RH, (D), (E), (F) are 3% RH, (G), (H), (I) are 20% RH and (J), (K) are 70% RH. MED1, 2, 4 refers to the Mean Escape Depth (Figure 2) of 0.9, 1.0 and 1.4 respectively.

Table S3: Deconvolution of XPS Cl 2p photoemission spectra.

**Cl 2p 0%RH**	Location BE Cl $2p_{3/2}$ (eV)	Location BE Cl $2p_{1/2}$ (eV)	Split (eV)	Height Cl $2p_{3/2}$	Height Cl $2p_{1/2}$	Area Cl $2p_{3/2}$	Area Cl $2p_{1/2}$	Width Cl $2p_{3/2}$	Width Cl $2p_{1/2}$
**MED1**	197.7	199.3	1.6	46359	24078	61627	32008	0.75	0.75
**MED2**	198.33	199.93	1.6	65752	32339	90903	44709	0.78	0.78
**MED4**	197.2	198.8	1.6	54845	27550	79712	40042	0.82	0.82
**Cl 2p 3%RH**	Location BE Cl $2p_{3/2}$ (eV)	Location BE Cl $2p_{1/2}$ (eV)	Split (eV)	Height Cl $2p_{3/2}$	Height Cl $2p_{1/2}$	Area Cl $2p_{3/2}$	Area Cl $2p_{1/2}$	Width Cl $2p_{3/2}$	Width Cl $2p_{1/2}$
**MED1**	200.7	202.3	1.6	62780	31621	83456	42035	0.75	0.75
**MED2**	198.38	199.98	1.6	129000	66310	178340	91675	0.78	0.78
**MED4**	198.78	200.38	1.6	229520	118350	333590	172010	0.82	0.82
**Cl 2p 20%RH**	Location BE Cl $2p_{3/2}$ (eV)	Location BE Cl $2p_{1/2}$ (eV)	Split (eV)	Height Cl $2p_{3/2}$	Height Cl $2p_{1/2}$	Area Cl $2p_{3/2}$	Area Cl $2p_{1/2}$	Width Cl $2p_{3/2}$	Width Cl $2p_{1/2}$
**MED1**	200.41	202.01	1.6	51863	26042	68944	34619	0.75	0.75
**MED2**	198.26	199.86	1.6	57735	28168	77773	37944	0.76	0.76
**MED4**	198.47	200.07	1.6	77539	39040	107200	53973	0.78	0.78
**Cl 2p 70%RH**	Location BE Cl $2p_{3/2}$ (eV)	Location BE Cl $2p_{1/2}$ (eV)	Split (eV)	Height Cl $2p_{3/2}$	Height Cl $2p_{1/2}$	Area Cl $2p_{3/2}$	Area Cl $2p_{1/2}$	Width Cl $2p_{3/2}$	Width Cl $2p_{1/2}$
**MED1**	199.1	200.7	1.6	22307	11594	28467	14796	0.72	0.72
**MED2**	198.2	199.8	1.6	45534	23080	58109	29454	0.72	0.72
**MED4**	198.43	200.03	1.6	60966	31259	81045	41554	0.75	0.75

Table S4: Deconvolution of XPS Mg 2p photoemission spectra.

**Mg 2p 0%RH**	Location BE Mg $2p_{3/2}$ (eV)	Location BE Mg $2p_{1/2}$ (eV)	Split (eV)	Height Mg $2p_{3/2}$	Height Mg $2p_{1/2}$	Area Mg $2p_{3/2}$	Area Mg $2p_{1/2}$	Width Mg $2p_{3/2}$	Width Mg $2p_{1/2}$
**MED1**	50.68	51.32	0.64	4974.9	2431.6	5907.9	2887.6	0.67	0.67
**MED2**	50.46	51.1	0.64	14700	7221.7	17718	8704	0.68	0.68
**MED4**	52.01	52.65	0.64	16086	7863.9	19959	9756.8	0.7	0.7
**Mg 2p 3%RH**	Location BE Mg $2p_{3/2}$ (eV)	Location BE Mg $2p_{1/2}$ (eV)	Split (eV)	Height Mg $2p_{3/2}$	Height Mg $2p_{1/2}$	Area Mg $2p_{3/2}$	Area Mg $2p_{1/2}$	Width Mg $2p_{3/2}$	Width Mg $2p_{1/2}$
**MED1**	50.28	50.92	0.64	5691.8	2897.4	6759.2	3440.8	0.67	0.67
**MED2**	50.19	50.83	0.64	25578	12680	30829	15283	0.68	0.68
**MED4**	47.9	48.54	0.64	22537	11165	27962	13852	0.7	0.7
**Mg 2p 20%RH**	Location BE Mg $2p_{3/2}$ (eV)	Location BE Mg $2p_{1/2}$ (eV)	Split (eV)	Height Mg $2p_{3/2}$	Height Mg $2p_{1/2}$	Area Mg $2p_{3/2}$	Area Mg $2p_{1/2}$	Width Mg $2p_{3/2}$	Width Mg $2p_{1/2}$
**MED1**	50.55	51.19	0.64	5443.9	2704.7	6464.9	3212	0.67	0.67
**MED2**	50.17	50.81	0.64	14603	7339.2	17600	8845.7	0.68	0.68
**MED4**	48.16	48.8	0.64	16320	8077.3	20249	10022	0.7	0.7
**Mg 2p 70%RH**	Location BE Mg $2p_{3/2}$ (eV)	Location BE Mg $2p_{1/2}$ (eV)	Split (eV)	Height Mg $2p_{3/2}$	Height Mg $2p_{1/2}$	Area Mg $2p_{3/2}$	Area Mg $2p_{1/2}$	Width Mg $2p_{3/2}$	Width Mg $2p_{1/2}$
**MED1**	50.09	50.73	0.64	8580	4202.8	10189	4991	0.67	0.67
**MED2**	49.3	49.94	0.64	16260	8051.7	20174	9989.9	0.7	0.7
**MED4**									

Table S5: Deconvolution of XPS S 2p photoemission spectra.

**S 2p 0%RH**	Location BE S $2p_{3/2}$ (eV)	Location BE S $2p_{1/2}$ (eV)	Split (eV)	Height S $2p_{3/2}$	Height S $2p_{1/2}$	Area S $2p_{3/2}$	Area S $2p_{1/2}$	Width S $2p_{3/2}$	Width S $2p_{1/2}$
**MED1**	167.46	168.66	1.2	17993	9298.1	22962	11866	0.72	0.72
**MED2**	168.89	170.09	1.2	37786	18843	50901	25382	0.76	0.76
**MED4**	169.67	170.87	1.2	77659	38094	111490	54691	0.81	0.81
**S 2p 3%RH**	Location BE S $2p_{3/2}$ (eV)	Location BE S $2p_{1/2}$ (eV)	Split (eV)	Height S $2p_{3/2}$	Height S $2p_{1/2}$	Area S $2p_{3/2}$	Area S $2p_{1/2}$	Width S $2p_{3/2}$	Width S $2p_{1/2}$
**MED1**	171.2	172.4	1.2	12875	6407	16431	8176.4	0.72	0.72
**MED2**	168.85	170.05	1.2	31542	16181	42489	21796	0.76	0.76
**MED4**	169.17	170.37	1.2	69894	35307	100350	50690	0.81	0.81
**S 2p 20%RH**	Location BE S $2p_{3/2}$ (eV)	Location BE S $2p_{1/2}$ (eV)	Split (eV)	Height S $2p_{3/2}$	Height S $2p_{1/2}$	Area S $2p_{3/2}$	Area S $2p_{1/2}$	Width S $2p_{3/2}$	Width S $2p_{1/2}$
**MED1**	170.78	171.98	1.2	10378	5154.4	13243	6577.9	0.72	0.72
**MED2**	168.7	169.9	1.2	24872	12548	33504	16902	0.76	0.76
**MED4**	168.87	170.07	1.2	17594	8875.7	25260	12743	0.81	0.81
**S 2p 70%RH**	Location BE S $2p_{3/2}$ (eV)	Location BE S $2p_{1/2}$ (eV)	Split (eV)	Height S $2p_{3/2}$	Height S $2p_{1/2}$	Area S $2p_{3/2}$	Area S $2p_{1/2}$	Width S $2p_{3/2}$	Width S $2p_{1/2}$
**MED1**	169.65	170.85	1.2	12285	6284	15242	7796.6	0.7	0.7
**MED2**	168.61	169.81	1.2	20875	10594	27381	13896	0.74	0.74
**MED4**	168.9	170.1	1.2	25991	12852	35012	17313	0.76	0.76

Table S6: Deconvolution of XPS Na 2s photoemission spectra.

**Na 2s 0%RH**	Location (eV)	Height	Area	Width
**MED1**	63.636	14207	16871	0.67
**MED2**	64.385	11341	14071	0.7
**MED4**	65.119	31647	42070	0.75
**Na 2s 3%RH**	Location (eV)	Height	Area	Width
**MED1**	63.698	16238	19283	0.67
**MED2**	63.375	13375	16667	0.70305
**MED4**	61.012	50471	67093	0.75
**Na 2s 20%RH**	Location (eV)	Height	Area	Width
**MED1**	63.461	7494	9032.2	0.68
**MED2**	63.386	14203	17622	0.7
**MED4**	61.149	29580	38273	0.73
**Na 2s 70%RH**	Location (eV)	Height	Area	Width
**MED1**	63.109	9811.6	11019	0.63362
**MED2**	62.058	19930	22457	0.6357
**MED4**				

Table S7: Deconvolution of XPS O 1s photoemission spectra.

**O 1s 0%RH**	Location BE SO_4^{2-} (eV)	Location BE H_2O_{sur} (eV)	Height SO_4^{2-}	Height H_2O_{sur}	Split BE H_2O_{sur} and SO_4^{2-} (eV)	Area SO_4^{2-}	Area H_2O_{sur}	Width SO_4^{2-}
**MED1**	530.7	531.95	232760	103080	1.25	408420	180870	0.99
**MED2**	531.7	532.95	295540	131100	1.25	544780	241670	1.04
**MED4**	534.95	536.2	186750	121510	1.25	410440	267060	1.24
**O1s 3%RH**	Location BE SO_4^{2-} (eV)	Location BE H_2O_{sur} (eV)	Location BE H_2O_{vap} (eV)	Height SO_4^{2-}	Height H_2O_{sur}	Height H_2O_{vap}	Split BE H_2O_{sur} and SO_4^{2-} (eV)	Split BE H_2O_{vap} and H_2O_{sur} (eV)
**MED1**	531.15	532.35	533.93	65440	112880	177060	1.2	1.58
**MED2**	531.7	532.95	534.45	86873	141350	104260	1.25	1.5
**MED4**	533.3	534.6	536.2	73029	123770	31029	1.3	1.6
**O 1s 20%RH**	Location BE SO_4^{2-} (eV)	Location BE H_2O_{sur} (eV)	Location BE H_2O_{vap} (eV)	Height SO_4^{2-}	Height H_2O_{sur}	Height H_2O_{vap}	Split BE H_2O_{sur} and SO_4^{2-} (eV)	Split BE H_2O_{vap} and H_2O_{sur} (eV)
**MED1**	531.45	532.65	534.24	85740	153530	180170	1.2	1.59
**MED2**	531.6	532.8	534.33	83902	149720	119260	1.2	1.53
**MED4**	532.9	534.25	535.85	74312	121840	34679	1.35	1.6
**O 1s 70%RH**	Location BE SO_4^{2-} (eV)	Location BE H_2O_{sur} (eV)	Location BE H_2O_{vap} (eV)	Height SO_4^{2-}	Height H_2O_{sur}	Height H_2O_{vap}	Split BE H_2O_{sur} and SO_4^{2-} (eV)	Split BE H_2O_{vap} and H_2O_{sur} (eV)
**MED1**	531.6	532.75	534.56	84839	127520	126230	1.15	1.81
**MED2**	531.58	532.79	534.56	86511	171780	104300	1.21	1.77
**MED4**	532.64	533.9	535.5	73777	106300	30199	1.26	1.6

**O 1s 0%RH**	Width H_2O_{sur}
**MED1**	0.99
**MED2**	1.04
**MED4**	1.24

**O1s 3%RH**	Area SO_4^{2-}	Area H_2O_{sur}	Area H_2O_{vap}	Width SO_4^{2-}	Width H_2O_{sur}	Width H_2O_{vap}
**MED1**	120630	208070	282450	1.04	1.04	0.9
**MED2**	175540	285610	184800	1.14	1.14	1
**MED4**	173450	293970	60497	1.34	1.34	1.1
**O 1s 20%RH**	Area SO_4^{2-}	Area H_2O_{sur}	Area H_2O_{vap}	Width SO_4^{2-}	Width H_2O_{sur}	Width H_2O_{vap}
**MED1**	158050	283010	271440	1.04	1.04	0.85
**MED2**	162100	289250	190240	1.09	1.09	0.9
**MED4**	163330	267800	61468	1.24	1.24	1
**O 1s 70%RH**	Area SO_4^{2-}	Area H_2O_{sur}	Area H_2O_{vap}	Width SO_4^{2-}	Width H_2O_{sur}	Width H_2O_{vap}
**MED1**	148870	223760	167810	0.99	0.99	0.75
**MED2**	159470	316640	147900	1.04	1.04	0.8
**MED4**	162150	233640	53526	1.24	1.24	1
